# Hepatocellular Carcinoma: Latest Research in Pathogenesis, Detection and Treatment

**DOI:** 10.3390/ijms241512224

**Published:** 2023-07-31

**Authors:** Beatrice Foglia, Cristian Turato, Stefania Cannito

**Affiliations:** 1 Department of Clinical and Biological Sciences, University of Turin, 10125 Turin, Italy; beatrice.foglia@unito.it; 2 Department of Molecular Medicine, University of Pavia, 27100 Pavia, Italy; cristian.turato@unipv.it

The most common form of primary liver malignancy is hepatocellular carcinoma (HCC). HCC, evolving in the setting of chronic liver disease (CLD) and cirrhosis, accounted globally for 906,000 new cases and 830,000 deaths in 2020, ranking it as the sixth most commonly diagnosed cancer and the third leading cause of cancer death, with a rapidly increasing trend [1]. In 2023, the North American Association of Central Cancer Registries estimates 41,210 new HCC cases diagnosed in the US, an incidence rate that has tripled over the past four decades, even though since 2015, the increase has stabilized [2].

Being an extremely heterogeneous tumor, also because of the different etiologies concurring with its development, HCC still retains an urgent need for new knowledge in the pathogenetic and therapeutic fields as well as in diagnostic, preventive, and personalized medicine. In this regard, it’s particularly interesting to note that HCC incidence and mortality rates increase in men worldwide, becoming 2 to 3 times higher compared to women regardless of disease etiology [1,2]. Montgomery et al. [3] have carefully explored the subject of sexual dimorphism in HCC, focusing their attention on many aspects linked to androgen receptor (AR) expression in the liver. Their review shows that AR mRNA or protein levels alone, correlating positively with improved survival, are insufficient to explain sexual dimorphism in HCC. On the other hand, AR activity should be a more reliable measure because it correlates with poor HCC outcomes, as demonstrated by the work of Ma et al. [4], where hepatic AR knockout (but not testosterone levels) in mice is responsible for impairment in carcinogen-mediated HCC progression. Interestingly, focusing on the AR-axis as a therapeutic target, the use of routine and effective approaches deployed in prostate cancer has thus far not elicited a response in HCC. This could probably be due to androgen-independent AR overexpression inducers like mTOR signaling, dysregulated lipogenesis, and constitutively active ligand-independent AR splice variants. Thus, additional work is needed to better understand the influence of androgen-independent AR inducers and their potential for HCC-specific action.

Another important factor influencing HCC heterogeneity is the tumor microenvironment (TME), which is built upon complex structures and cellular and molecular components, including, among others, cancer-associated fibroblasts (CAFs). Fibroblasts play a key role in HCC progression, especially considering about 80% of HCCs arise in a cirrhotic liver. CAFs modulate TME through various mechanisms influencing cancer fate and treatment responses. In this issue, Akkiz [5] recapitulates the various aspects of CAF origin and activation mechanisms, their impact on HCC progression, and the efficacy of anticancer approaches. From this comprehensive analysis, it emerges that CAFs are critical for HCC development and progression, being involved in several processes (such as extracellular matrix-ECM-remodeling, maintenance of stemness, angiogenesis, metabolic modulation, immune responses, promotion of cancer cell proliferation, invasion, and therapeutic resistance), suggesting that a big effort needs to be performed in better elucidating the signaling pathways that mediate crosstalk between CAFs and cancer cells.

CAFs and cancer cells mediate the modulation of the metabolic landscape in tumors. In their review, Tian et al. [6] have summarized the mechanisms by which the PI3K/AKT/mTOR pathway can induce HCC metabolic reprogramming. They show that AKT/mTOR signaling increases aerobic glycolysis, lactate metabolism, and induction of 6-phosphogluconate dehydrogenase (G6PD) activity, favoring the pentose phosphate pathway (PPP) and thus promoting the growth of cancer cells. This pathway can also increase the transcription of key genes involved in fatty-acid synthesis in HCC cells like SREBP1/2 in addition to pump glutamine metabolism and pyrimidine synthesis to promote proliferation. Moreover, they have discussed the contribution of reactive oxygen species (ROS) production in modulating PI3K/AKT/mTOR and how these signaling pathways can influence tumor immune response. Since the PI3K/AKT/mTOR pathway is critically involved in several cellular processes, the authors provided an interesting point of view on the ongoing clinical trials based on the inhibition of the PI3K/AKT/mTOR pathway in HCC in addition or combination with other drugs.

Another interesting study by Shueng et al. [7] explores sorafenib resistance from a metabolic point of view, providing evidence that suppression of fatty acid synthesis in an in vitro setting can overcome the resistant phenotype. In fact, using the fatty acid synthase (FASN) inhibitor, orlistat, the authors showed that combination treatment strongly enhanced sorafenib cytotoxicity by shifting metabolism and promoting cell death.

Another fundamental aspect of metabolic reprogramming of cancer cells is the consequent modulation of immune responses. In our review composed of this issue [8], we have proposed a tracking shot of what is currently known about the alterations of glucose, fatty acid, amino acid, and glutamine metabolism and their influence on HCC microenvironment and particularly on the different immune cell populations, potentially favoring the tumor escape from immunosurveillance. The review also highlights the peculiarity emerging concerning different etiology and discusses the future perspective in this field. In fact, there is still much to learn on how HCC remodels the immunosuppressive TME, limiting the efficacy of immunotherapy. Resolving the mechanism leading HCC cells to evade immune surveillance and acquire resistance to immune checkpoint inhibitors (ICIs) will surely be a milestone, as it will provide new valuable diagnostic markers and therapeutic targets for the prevention and the fight against HCC. In this regard, two remarkable studies by Peng et al. [9] and Hammad et al. [10] have provided data on promising candidate biomarkers for the diagnosis, prognosis, and response to immunotherapy in HCC. The first work revealed that zinc finger protein 385A (ZNF385A) and 346 (ZNF346) both correlate with tumor immunosuppression and inflammation and with a poor overall prognosis of HCC patients [9]. In contrast, the second states that leukocyte-associated immunoglobulin-like receptor-1 (LAIR-1), an inhibitory checkpoint expressed on cytotoxic T cells (Tcs), was found overexpressed in an Egyptian HCV+ cohort of HCC patients and correlated with tumor marker AFP, insulin resistance and inflammation prognostic ratios/indices [10]. In addition, Tsai et al. [11] reported that the serum levels of large HBV surface protein (LHBS) expression increased in an HBV+ cohort of HCC patients, where it plays a specific role in HCC progression and correlates with cirrhosis and worse disease-free and overall survival rates, representing an intriguing non-invasive biomarker for HCC patients with a worse prognosis after surgery.

As demonstrated by the various studies collected in this Special Issue, knowledge of the pathological mechanisms that lead to tumor development and progression is essential for correctly understanding both the elements of prevention and planning of therapeutic strategies. In this regard, in vitro research is an exceptionally flexible and useful tool, but it still retains a series of gaps and problems that must be resolved. Great strides have been made in recent years with the use of 3D culture techniques, which have made it possible to better summarize the features of tissues and organs in the tumor field. In this regard, De Siervi and Turato [12] provide an elegant discussion of the advantages offered by spheroids, scaffold-based 3D systems, 3D bioprinting, and organoids, especially concerning applications in regenerative medicine and drug discovery, offering an exhaustive overview of the different protocols currently used. An interesting example of how 3D cultures can provide a more detailed and precise view of cell behavior is given by the work of Gao et al. [13]. In this original work, the authors provided first-time evidence of a specific role of SPRED2, which was found to be downregulated in HCC patients, demonstrating that endogenous SPRED2 negatively regulates the ability of HCC cells to duplicate, migrate, and invade. Moreover, comparing 2D and 3D models, they revealed the high impact of endogenous SPRED2 contribution in suppressing epithelial–mesenchymal transition and the acquisition of stemness. The use of spheroids was again proved to be a remarkable tool in the work of Scagliola et al. [14], where the combination of in vivo and in vitro studies has given a profound insight into the role of eukaryotic initiation factor 6 (eIF6), a translation controller that exacerbates lipid accumulation through a strong increase in FASN levels, contributing in nonalcoholic fatty liver disease (NAFLD) evolution towards HCC. They found that heterozygosity for eIF6 in mice reduced the formation of HCC nodules and that eIF6 depletion or pharmacological inhibition reduces the growth of HCC spheroids.

Ultimately, the authors of the 11 articles and reviews have provided a new, lucid, and innovative vision of all the essential aspects of dealing with pathologies such as HCC, that is pathological mechanisms, prevention, and therapy.
